# Relatedness within and between Agta residential groups

**DOI:** 10.1017/ehs.2021.46

**Published:** 2021-09-22

**Authors:** Mark Dyble, Andrea Bamberg Migliano, Abigail E. Page, Daniel Smith

**Affiliations:** 1 Department of Anthropology, University College London, London, UK; 2 Department of Anthropology, University of Zurich, Zurich, Switzerland; 3 Department of Population Health, London School of Hygiene and Tropical Medicine, London WC1E 7HT, UK; 4 Bristol Medical School (PHS), University of Bristol, Bristol, UK.

**Keywords:** hunter–gatherers, relatedness, social organisation, Agta, kinship dynamics, affinal kin

## Abstract

Theoretical models relating to the evolution of human behaviour usually make assumptions about the kinship structure of social groups. Since humans were hunter–gatherers for most of our evolutionary history, data on the composition of contemporary hunter–gatherer groups has long been used to inform these models. Although several papers have taken a broad view of hunter–gatherer social organisation, it is also useful to explore data from single populations in more depth. Here, we describe patterns of relatedness among the Palanan Agta, hunter–gatherers from the northern Philippines. Across 271 adults, mean relatedness to adults across the population is *r =* 0.01 and to adult campmates is *r =* 0.074, estimates that are similar to those seen in other hunter–gatherers. We also report the distribution of kin across camps, relatedness and age differences between spouses, and the degree of shared reproductive interest between camp mates, a measure that incorporates affinal kinship. For both this this measure (*s*) and standard relatedness (*r*), we see no major age or sex differences in the relatedness of adults to their campmates, conditions that may reduce the potential for conflicts of interest within social groups.

## Graphical abstract

**Figure:**
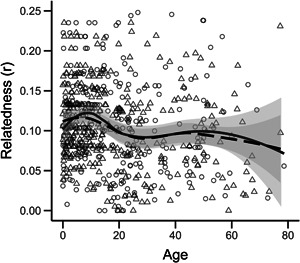

**Social media summary:** Agta hunter–gatherers do not experience age- or sex-related differences in relatedness to their campmates.

## Introduction

Across group-living mammals there is a clear association between patterns of social behaviour and the kinship structure of groups, with cooperative behaviours being most highly developed in groups in which the average degree of relatedness is high (Lukas & Clutton-Brock, 2018). Broadly, this is because individuals derive indirect fitness benefits through altruistic behaviours directed towards related individuals; altruism is more likely to be favoured by selection when it occurs among kin (Gardner et al., 2011; Hamilton, 1964; Lehmann & Keller, 2006; West et al., 2021). In human evolution, all models for the evolution of sociality make some kind of assumption about population structure and relatedness. For instance, differing views about the kinship structure and boundedness of human groups in evolutionary history are embedded in debates about the relative importance of kin vs. group selection in human evolution (Birch, 2019). In some cases, assumptions about relatedness are explicit while in other models relatedness and population structure play an important, but unrecognised role (Dyble, 2021). Indeed, many models that have claimed to demonstrate the evolution of cooperation through processes other than kin selection have, in fact, unwittingly rediscovered the role of relatedness (Liao et al., 2015; Kay et al., 2020).

Given the role of relatedness in the evolution of social behaviour, it is of interest and importance to establish what kind of relatedness and population structure may have been commonplace in human evolutionary history. In order to establish this, it has been typical to reason by ethnographic analogy and to examine the social organisation of contemporary hunter–gatherer societies who probably face many of the same economic and ecological opportunities and constraints as foragers in the past. What patterns of social organisation are typical among contemporary hunter–gatherers? Although there are potential pitfalls to reasoning by ethnographic analogy (Marlowe, 2005; Page & French, 2020) and delayed-return foraging populations are probably underrepresented in the ethnographic record (Singh & Glowacki, 2021), generalising across the available data we can see that contemporary hunter–gatherers typically live in camps in which the majority of co-resident adults are distantly related or unrelated (Hill et al., 2011; Walker & Bailey, 2014; Walker, 2014) and in which households move often between groups and there are not strong sex biases in residence and dispersal (Dyble et al., 2015; Hill et al., 2011; Wood & Marlowe, 2011). Across data compiled in Walker (2014), the mean group relatedness across 34 hunter–gatherer populations (as measured by Wright's coefficient of relatedness, *r*) was 0.080. These estimates situate humans at relatively low relatedness compared with other highly social mammals living in similarly sized groups (Dyble & Clutton-Brock, 2020) and far from cooperatively breeding species with which humans are sometimes compared (e.g. *r =* 0.34 among meerkats (Duncan et al., 2019), *r =* 0.46 in Damaraland mole-rats (Burland et al., 2002) and *r =* 0.27 among African Wild Dogs (Girman et al., 1997)). In the more politically stratified hunter–gatherer societies that are argued to be underrepresented in ethnographic data (Singh & Glowacki, 2021), the larger group sizes mean that relatedness within groups would probably be lower still, in accordance with the negative relationship between relatedness and group size shown in Walker (2014).

As well as establishing the average degree of relatedness among groups, it is also important to understand age and sex differences in the relatedness of individuals to their group since asymmetries in relatedness to the group may lead to conflicts of interest among group members with consequences for social behaviour (Croft et al., 2021). For example, Cant and Johntone (2008) suggest that the evolution of menopause in humans can be partly explained by an asymmetry in relatedness to offspring between older and younger women that results from female dispersal. Similarly, kin selection modelling by Micheletti et al. (2017) suggests that sex-differences in relatedness to the group may lead to intrafamilial conflicts of interest with respect to the fitness benefits of engaging in intergroup violence and differences in the benefits of altruistic behaviour towards groupmates Micheletti, Ruxton & Gardner (2020). Assessing the plausibility of such hypotheses for human social evolution requires more than average relatedness; we also need detailed data on age and sex differences in residence patterns and relatedness to group mates, patterns described by Croft et al. (2021) as ‘kinship dynamics’.

Another important dimension of relatedness is the distribution of kin across communities and the degree of relatedness between groups. Among hunter–gatherers, having extensive networks of kin across space has been argued to be beneficial to individuals because the ability to move between camps containing kin can provide insurance against local environmental failure (Wiessner, 2002) and provide opportunities for marrying outside of one's group (Kramer et al., 2017). At a group level, hunter–gatherer social organisation has also been suggested to facilitate cumulative cultural exchange (Hill et al., 2014; Migliano et al., 2020) and, more generally, having tolerant relationships with kin in neighbouring groups has been argued to allow humans to form large multilevel societies (Bird et al., 2019; Chapais, 2008; Grueter et al., 2020). Although the benefits of cultural exchange and multilevel sociality can be considered emergent or ‘group-level’ benefits, the social organisation that promotes them can be plausibly explained as a by-product of individual-level residential decision making (Dyble et al., 2015).

Here, we provide a detailed quantitative description of the relatedness within and between residential groups (‘camps’) of the Palanan Agta, a community of foragers from the northern Philippines described below. We explore patterns of relatedness within and between camps, across age and sex, and between spouses. We also assess whether relatedness across groups varies with the degree of population turnover (a proxy for mobility) or engagement in non-foraging economic activities. We find that in camp size and within-camp relatedness the Agta are comparable with previously described immediate-return foraging populations, that there are no age or sex differences in relatedness to the camp, that relatedness between residential camps is low and negatively correlated with distance, and that reduced mobility and engagement in foraging are not correlated with within-group relatedness. These findings provide a detailed quantitative portrait of relatedness among the Agta and show that a bilocal residence system in which either sex may disperse can produce conditions that reduce the potential for conflicts of interest within social groups.

## Methods

### Ethnographic background

The Agta number around 10,000 people and live in northeastern Luzon, Philippines. The Palanan Agta are a subpopulation of the Agta and live in the municipality of Palanan in Isabela province. As described elsewhere (Minter, 2008), the Palanan Agta are presently engaged in a mixed economy dominated by foraging and wet-rice agricultural labour, albeit with substantial differences between camps, with some camps still engaged almost entirely in foraging and others involved largely in agriculture (Dyble et al., 2019; Minter, 2008). Across the 10 camps included in this study for which time budget data were available, foraging as a proportion of all out-of-camp camp work ranged from 19.5 to 100%, averaging 67%. Foraging among the Agta consists of hunting, spearfishing (both riverine and marine), the gathering of wild plant foods and honey collecting. An estimated 28% of foraged food by weight is traded, usually for rice, increasing the calorific return by ~3 times (Dyble et al., 2019; Minter, 2008). Food is shared extensively between households, although most smaller items are shared within small clusters of closely related households (Dyble et al., 2016; D. Smith et al., 2019). While many Palanan Agta households and camps remain highly mobile, others have become more sedentary (Page et al., 2016; D. Smith et al., 2016). Across the 11 camps included in this study for which data on camp stability can be estimated, this varied from 0.12 to 0.79 and averaged 0.51, where 1 indicates no change in camp membership between our visits and 0 indicates complete turnover.

Previous work on the Palanan Agta has reported average camp size of around 20 adults within which ~25% of adult dyads are primary kin (Dyble et al., 2015) and in which almost all adults have a kinship connection to at least one other adult in the camp (Dyble, 2020). This is consistent with Griffin's (1984) report that the Agta need some kind of kinship tie to an existing camp member in order to join a camp. Mean relatedness among Agta camps has been estimated for the Casiguran Agta as 0.053 (Walker, 2014) and for a smaller sample of Palanan Agta communities than in the present study as *r =* 0.12 (Dyble et al., 2019), but note this estimate is inflated in that it is the mean relatedness of camps rather than of individuals to their campmates (see below). Although in principle the Agta prohibit marriage between individuals who can refer to one another by a kinship term (Early & Headland, 1998; Minter, 2008), exceptions to this are sometimes made (Headland, 1987).

### Estimating relatedness

Data on relatedness among the Agta were collected during fieldwork in 2013–2014 during which MD, DS and AEP visited all Agta communities in the municipality of Palanan. In every camp visited, we conducted in-depth genealogical interviews with all adults. These interviews were cross-checked and compiled into a genealogy within which our sampled individuals have a mean depth of 3.34 generations (SD = 1.26) and a maximum depth of six generations. As shown by Pemberton (2008), genealogies with a depth of three generations will capture the majority of variation in relatedness. We then used the genealogical data to estimate the dyadic relatedness of all individuals using functions from the *pedigree* and *kinship2* packages (Coster, 2012; Therneau et al., 2014). We estimated both genealogical relatedness (*r*), a commonly used measure (Wright, 1922), and shared reproductive interest (*s*) which measures the extent to which an individual will expect to be genetically related to another individual's future offspring, relative to their own offspring. Following Dyble et al. (2018) this is defined as *s* = (*r*_B_ + *r*_D_)/(1 + *r*_C_) where *r*_C_ is the relatedness of the ego to their spouse, *r*_B_ is the relatedness of ego to alter and *r*_D_ is the relatedness of ego to alter's spouse. In the absence of consanguineal marriages, *s* will be identical to *r* for consanguineal kin but will also capture the shared reproductive interests that exist among affinal kin (Dyble et al., 2018). For both *r* and *s*, we estimated the mean relatedness of individuals to their campmates unless otherwise stated. It is important to note that this is one of three ways in which relatedness could be aggregated. The first option is *r*_group_, which is where the relatedness between all group members is averaged separately for each group and then averaged across groups. The second option is *r*_individual_, where the average relatedness of each individual to their groupmates is calculated and then averaged across individuals. The third option is *r*_dyads_, which is the average relatedness of all co-resident dyads in the sample. The choice of estimate is relevant because where groups differ in size and larger groups are less closely related (as is typical), then *r*_dyads_ < *r*_individual_ < *r*_groups_. For example, imagine we have two communities: one with 20 individuals all related to each other by *r =* 0.1 and one with 100 individuals related to each other by 0.02. Mean relatedness within groups (*N =* 2) is 0.06, mean relatedness of individuals to their group (*N =* 120) is 0.033 and mean relatedness of co-resident dyads (*N =* 5080) is 0.023. All three of these measures have some theoretical relevance but *r*_individual_ is probably the most salient as it determines the indirect fitness benefits that an individual will derive for altruistic behaviours directed towards their group. Previous studies reporting relatedness in human communities are not always explicit about which estimate of relatedness is used although Koster et al. (2019) and Blurton-Jones (2016) imply use of *r*_individual_ and Walker (2014) is clear in using *r*_group_.

The total sample includes data on 615 individuals from 15 camps including 145 adult men and 126 adult women. These are the same 15 camps analysed in Dyble (2020) and include the 11 camps described in Dyble et al. (2015) as well as four camps visited in summer 2014 and not included in the 2015 paper. The categorical composition of these four additional camps is similar to the composition of the 11 camps set out in that paper, with 26.3% (262 of 1004) adult dyads in these four camps being consanguineal (i.e. genetic) kin (vs. 23.9% (924 of 3864) of dyads in Dyble et al., 2015).

### Other variables

Since the Agta do not necessarily know their age in calendar years, estimated age was taken as the mean from a probability distribution of the age of each individual produced by a Gibbs sampling Markov chain Monte Carlo algorithm described in Diekmann et al. (2017) that incorporates data from relative age lists produced by informants, broad estimated age brackets from researchers and some known ages that act as ‘anchors’. These estimates were available for all but eight individuals in the study population. These eight individuals were assigned simply as being adults or children. The locations of camps were taken by GPS readings or estimated using Google Earth and distances between camps calculated using functions from the *geodist* package. Camp stability was measured as in D. Smith et al. (2016) by noting the residents of a camp each time it was visited and calculating the amount of change in camp composition over time, with ‘1’ indicating no change in camp composition and ‘0’ representing complete turnover. Engagement in foraging was measured through the amount of time spent foraging as a proportion of all time spent working out of camp and was assessed using time budget data collected in 10 of the study camps according to the methods set out in Dyble et al. (2019).

## Results

**Figure 1.**
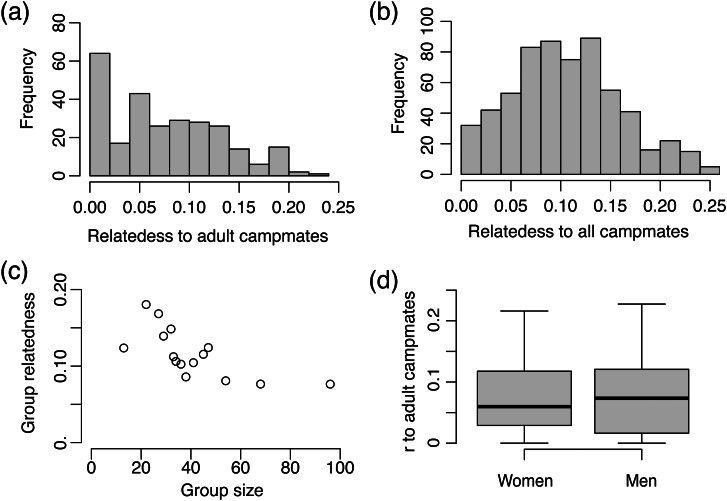
Relatedness (*r*) estimates among the Agta. (a) Histogram of the average relatedness of all 271 adults to their adult campmates; (b) histogram of the average relatedness of all 615 individuals to all their campmates including children; (c) average relatedness between all campmates plotted against camp size for each of the 15 camps; and (d) relatedness of adult women and men to their adult campmates.

Across 271 Agta adults, mean relatedness to all other adults in the population was *r =* 0.010 (SD = 0.006) and mean relatedness to adult campmates was *r =* 0.074 (SD = 0.058, Figure 1a). Mean camp size was 18.1 adults (SD = 8.63). Across the 15 camps, camp size was negatively correlated with intracamp relatedness between adults (*r =* −0.68, *p* = 0.005, Figure 1c). When relatedness was estimated as the average relatedness of all individuals to all campmates (i.e. including children), mean *r* was slightly higher at 0.095 (SD = 0.054, *N =* 615, Figure 1b). Mean relatedness of adults to children in their camp was 0.112 (SD = 0.072, *N =* 271), and mean relatedness between children was 0.117 (SD = 0.059, *N =* 344).

As set out above, affinal kin may have shared reproductive interests in the next generation and this can be measured with *s*, which between adults in the population was, on average, *s* = 0.016 (SD = 0.011) and between adults and their adult campmates was 0.154 (SD = 0.103). This means that for the average Agta adult, the future offspring of a randomly selected adult campmate is expected – in terms of identity by descent – to be 15% as related to them as they would be to their own future offspring. This, rather than relatedness, is arguably the most salient measure of the fitness benefits that individuals receive from altruism directed towards campmates (Dyble et al., 2018; cf. Daly & Perry, 2021).

Camp stability, an indicator of the degree of sedentism/mobility, was uncorrelated with camp relatedness, either as a simple correlation (*r =* −0.077, *p* = 0.82, *n* = 11) or as a partial correlation controlling for camp size (*r =* −0.20, *p* = 0.57). The proportion of out of camp work time that camp members engaged in foraging was correlated with camp relatedness (*r =* 0.64, *p* = 0.047), but this was not preserved in a partial correlation controlling for group size (*r =* 0.14, *p* = 0.71); camps engaged in more market-integrated activities tended to be larger but no more or less closely related than expected given their size.

### Sex and age differences in relatedness to the camp

**Figure 2.**
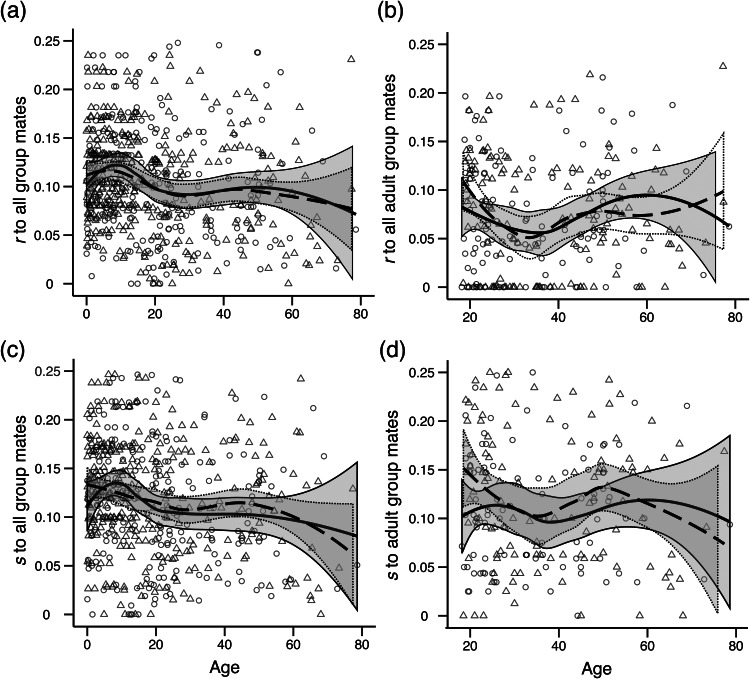
Relatedness and shared reproductive interest of individuals to campmates by age and sex for (a) *r* of all individuals to all campmates (including children), (b) *r* of adults to adult campmates, (c) *s* of all individuals to all campmates (including children) and (d) *s* of adults to adult campmates. Boys/men are triangles, girls/women are circles. Lines are LOESS curves with 0.5 sensitivity and grey bands show the standard error. Solid lines and solid bands are for girls/women and dashed lines and dotted bands are for boys/men.

We see no sex difference in the average relatedness of adults to their adult campmates: men, *r =* 0.076 (SD = 0.059, *N =* 145); women, *r =* 0.073 (SD = 0.058, *N =* 126); two-tailed permutation test, *p* = 0.694; Figure 1d). Similarly, we see no sex difference in the average degree of shared reproductive interest between adults and their adult campmates: men, *s* = 0.152 (SD = 0.097, *N =* 145); women, *s* = 0.155 (SD = 0.111, *N =* 126); two-tailed permutation test, *p* = 0.798). In contrast to many non-forager societies where relatedness varies with sex and age (Koster et al., 2019), our cross-sectional analysis suggests that relatedness to campmates remains relatively constant throughout adult life for both men and women (Figure 2a), albeit with a slight reduction in the relatedness of both men and women to their adult campmates from 20 to 30 years of age associated with dispersing to marry and a subsequent increase as adult children begin to be included as campmates (Figure 2b). Similar results are obtained for estimates of shared reproductive interest (Figure 2c and d).

### Distribution of kin across camps

**Figure 3.**
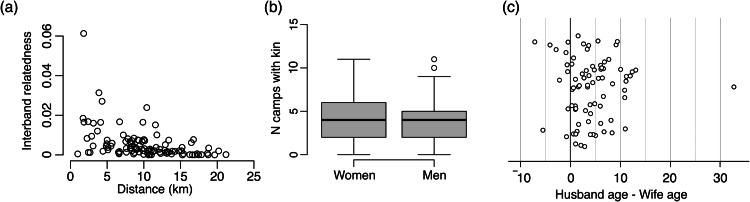
(a) Scatter plot showing the average adult relatedness and distance between each pair of camps (*N =* 105 pairs); (b) histogram of the distribution of number of camps containing consanguineal kin (*r* ≥ 0.0625) for women and men; and (c) jitter plot showing the estimated age differences between Agta spouses.

There is a negative correlation between the relatedness between adults from each pair of camps and the distances between those camps (Spearman *r =* −0.51, *p* < 0.001, Figure 3a). The combination of a near absence of close consanguineal marriage and the bilocal residence means that Agta kinship networks are relatively diffuse and, on average, adults had consanguineal kin of at least *r =* 0.0625 (equivalent to a cousin's child) in 4.07 of the 15 study camps (SD = 2.58, range 0–11, *N =* 271 adults). Note that many of these adults will also have kin in camps outside of the municipality where data collection was focused, such that the full extent of their kin network is much greater. Although women had, on average, kin living in a slightly larger number of camps (men, mean = 3.87, SD = 2.43, *N =* 145; women, mean = 4.29, SD = 2.74, *N =* 126, Figure 3b), this difference is not significant (permutations test, *p* = 0.088, Figure 3b). Affinal relationships also play a role in extending kinship networks across camps: by virtue of being able to reside with male and female kin of both the husband and wife, the average household can reside in 6.70 of the 15 camps (SD = 2.94, range 1–14, *N =* 158).

### Consanguinity and age differences between spouses

As noted above, the Agta have rules against marrying kin. Although Headland (1987) suggests that in practice these rules are pragmatically interpreted and Minter (2008) suggests that rates of consanguineous marriage (i.e. marriage between close genetic relatives) may be increasing, our data show that such marriages remain rare among the Palanan Agta. Of 80 marriages, 78 had no known shared genealogical ancestry, one couple were second cousins (*r =* 0.03125), and one couple were first cousins (*r =* 0.125; the husband's mother and wife's father are full siblings). As an additional analysis, we looked at the age differences between Agta spouses. Of the 78 marriages where we had an age estimate for both individuals, the husband was older in 67 marriages (86%) and the wife was older in 11 (14%) (Figure 3c). The average difference between husband age and wife age (calculated as husband age – wife age) was 4.29 years (SD = 5.31) and ranged from −7.1 to 32.7 years. This maximum difference of 32.7 years was between a 17-year-old woman and 50-year-old man and was a large outlier; the second largest difference was 13.1 years.

## Discussion

Here, we have provided a quantitative description of the relatedness structure within and between Agta residential camps. These data show sex equality in Agta residence, with no sex difference in either relatedness to the camp or the distribution of kin across residential camps. The results also show that, on average, the relatedness of adults to their camp mates does not change with age. This is in contrast to what is seen in populations in which one sex disperses and increases their relatedness to their group with age (Koster et al., 2019). From an inclusive fitness point of view, there are no asymmetries in the benefits that any sex or age class of individual will derive from cooperating with their campmates. This is significant because asymmetries in relatedness between men and women of different ages are a critical assumption in many models of social evolution (Croft et al., 2021). For example, Cant and Johnstone's (2008) model of reproductive conflict assumes that female-biased dispersal results in older women being more closely related to the children of younger women (their son's wives children) than younger women are to the children of older women (their husband's mother's children). Under these conditions and in the event of reproductive competition, younger women would be insensitive to costs felt by older women, potentially leading to early cessation of reproduction. The female-biased residence required for the evolution of menopause in this model is not seen in our data and is not typical of immediate-return hunter–gatherers more broadly (Hill et al., 2011).

The mean relatedness between adults across the whole population of the Palanan Agta of *r =* 0.01 is similar to that reported for the Hadza (Blurton Jones, 2016) and the average relatedness of adults to their adult campmates of 0.074 is also similar to previously described estimates for hunter–gatherers: Walker (2014) estimates a mean *r* of 0.080 across 34 hunter–gatherer groups and Burton-Jones estimates *r =* 0.0753 among the Hadza (Blurton Jones, 2016). What determines the relatedness of these groups? As we have argued previously (Dyble et al., 2015), group relatedness is likely to be constrained under bilocal residence, as seen among the Agta and bilocal hunter–gatherers who tend to live in groups of lower relatedness than horticultural or agricultural groups with sex-biased dispersal, even if controlling for group size (Dyble et al., 2015; Walker, 2014). More generally, important features of human demography and life history such as monotocy (the production of single offspring per pregnancy) and low reproductive skew are also predicted to reduce group relatedness (Dyble & Clutton-Brock, 2020). Whether our estimates of relatedness within groups are based on hunter–gatherers or other societies, these estimates situate humans at relatively low relatedness compared with other highly social mammals living in similarly sized groups (Dyble & Clutton-Brock, 2020) and similar to estimates from chimpanzees (Langergraber et al., 2007). As such, even if humans can be reasonably argued to be cooperative breeders (e.g. Hrdy, 2007; Kramer, 2010; Van Schaik & Burkart, 2010) then the evolutionary pathway that led to this is probably very different from the pathway to cooperative breeding in other mammals based on a high degree of within-group relatedness (Lukas & Clutton-Brock, 2012, 2018).

The low frequency of consanguineal marriage seen among the Palanan Agta is also typical of contemporary hunter–gatherer societies, and in contrast to the greater frequency of consanguineal marriage seen among agropastoralists (Bailey et al., 2014; Walker & Bailey, 2014). In combination with bilocal residence, these low rates of kin marriage mean that the Agta have diffuse networks of kin across camps of the kind that have been argued to have been important in buffering individuals against local environmental failure (Wiessner, 1977), in facilitating the exchange of goods and cultural ideas (Dyble, 2018; Hill et al., 2014; Migliano et al., 2020) and in increasing the availability of marriage partners in groups otherwise susceptible to random fluctuations in adult sex ratio (Kramer et al., 2017).

Overall, our results show that in terms of camp size, residence patterns and within-group relatedness, the Palanan Agta are similar to other immediate-return hunter–gatherer communities including the Ache, Hadza and Ju/’hoansi (Blurton Jones, 2016; Hill & Hurtado, 1996; Hill et al., 2011; Walker, 2014). On one hand, the similarities in social organisation between hunter–gatherer societies living on different continents and in diverse environments represent remarkable consistency, lending weight to the use of the ethnographic analogy to reconstruct the social organisation of foragers in the past and to the foundational assumption of human behavioural ecology that convergence in lifeways will occur in response to similar subsistence challenges. However, it is clear that contemporary immediate-return hunter–gatherer societies are not representative of *all* hunter–gatherers in the past because the existing sample may preferentially be seen in marginal environments unsuitable for farming (Cunningham et al., 2019; Marlowe, 2005; Porter & Marlowe, 2007), because a focus on immediate-return foragers neglects the more marked social and political complexity and inequality seen in many delayed-return and sedentary foragers, usually associated with defensible resources (Moreau, 2020; Ringen et al., 2021; E. A. Smith & Codding, 2021), and because contemporary hunter–gatherers have important relationships with neighbouring non-foraging groups and wider state societies that may have influenced their way of life (Headland et al., 1989; Lee & Guenther, 1991; Singh & Glowacki, 2021; Wilmsen, 1989). Therefore, even if the kind of social organisation seen among contemporary hunter–gatherers is a good model for some foraging societies in human evolutionary history, it is unlikely to have been some kind of ‘universal’ form. However, as our results demonstrate, when economic and environmental conditions do facilitate mobility and bilocal residence, individuals may vary very little in their relatedness to camp mates by either sex or age. Although relatedness is not the only determinant of the fitness benefits of cooperation, it plays an important role and a lack of asymmetry in relatedness may promote the egalitarian political systems seen among the Agta and many other immediate-return foraging societies (Boehm, 2009; Kelly, 2013; Woodburn, 1982).

## Data Availability

The data and code are available as Supplementary Materials.

## Supplementary material

For supplementary material accompanying this paper visit https://doi.org/10.1017/ehs.2021.46. click here to view supplementary material
